# Supplementary material for: Causal Relationship between Aging and Anorexia Nervosa: A White-Matter-Microstructure-Mediated Mendelian Randomization Analysis
Source: Biomedicines. 2024 Aug 16;12(8):1874. doi: 10.3390/biomedicines12081874 (PMC11351342; doi:10.3390/biomedicines12081874)
Supplement: Supplementary file 1 [file biomedicines-12-01874-s001.zip › Supplementary Figure/Figure S4/Singe SNP plot of MR analysis for TL to ubm-b-1740.pdf]

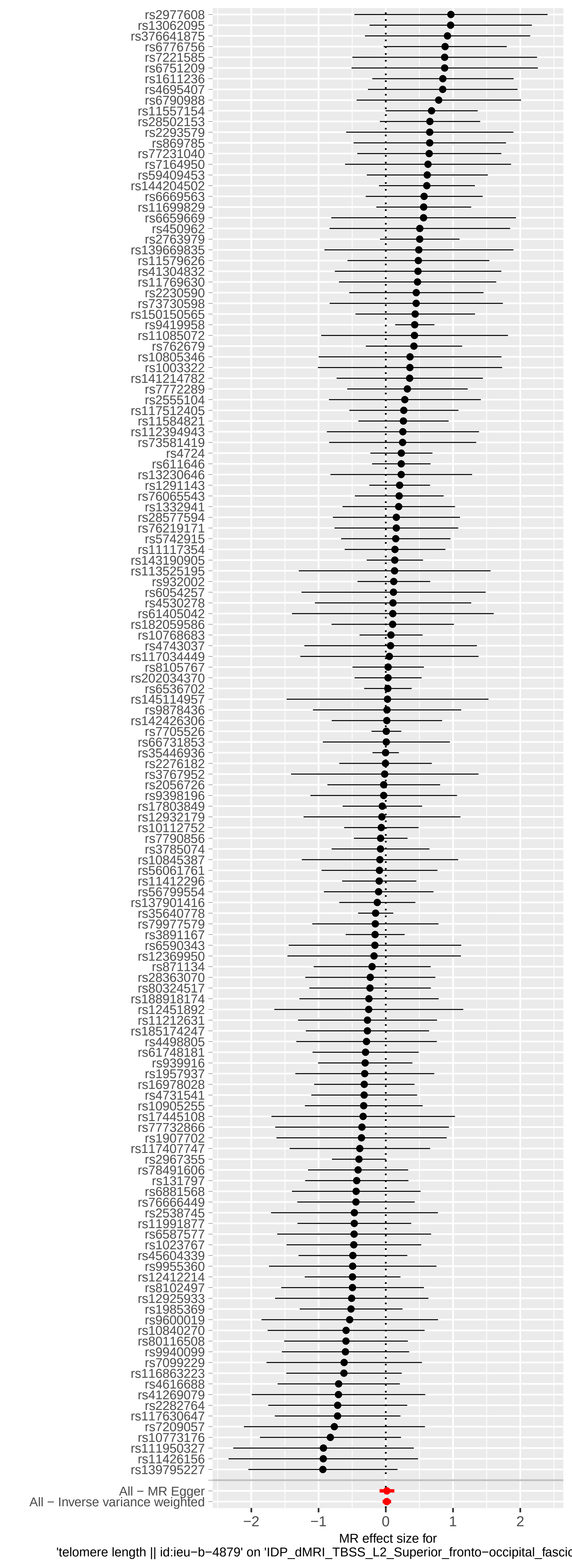
rs2977608
rs13062095
rs376641875
rs6776756
rs7221585
rs6751209
rs1611236
rs4695407
rs6790988
rs11557154
rs28502153
rs2293579
rs869785
rs77231040
rs7164950
rs59409453
rs144204502
rs6669563
rs11699829
rs6659669
rs450962
rs2763979
rs139669835
rs11579626
rs41304832
rs11769630
rs2230590
rs73730598
rs150150565
rs9419958
rs11085072
rs762679
rs10805346
rs1003322
rs141214782
rs7772289
rs2555104
rs117512405
rs11584821
rs112394943
rs73581419
rs4724
rs611646
rs13230646
rs1291143
rs76065543
rs1332941
rs28577594
rs76219171
rs5742915
rs11117354
rs143190905
rs113525195
rs932002
rs6054257
rs4530278
rs61405042
rs182059586
rs10768683
rs4743037
rs117034449
rs8105767
rs202034370
rs6536702
rs145114957
rs9878436
rs142426306
rs7705526
rs66731853
rs35446936
rs2276182
rs3767952
rs2056726
rs9398196
rs17803849
rs12932179
rs10112752
rs7790856
rs3785074
rs10845387
rs56061761
rs11412296
rs56799554
rs137901416
rs35640778
rs79977579
rs3891167
rs6590343
rs12369950
rs871134
rs28363070
rs80324517
rs188918174
rs12451892
rs11212631
rs185174247
rs4498805
rs61748181
rs939916
rs1957937
rs16978028
rs4731541
rs10905255
rs17445108
rs77732866
rs1907702
rs117407747
rs2967355
rs78491606
rs131797
rs6881568
rs76666449
rs2538745
rs11991877
rs6587577
rs1023767
rs45604339
rs9955360
rs12412214
rs8102497
rs12925933
rs1985369
rs9600019
rs10840270
rs80116508
rs9940099
rs7099229
rs116863223
rs4616688
rs41269079
rs2282764
rs117630647
rs7209057
rs10773176
rs111950327
rs11426156
rs139795227
All − MR Egger
All − Inverse variance weighted
−2
−1
0
1
2
MR effect size for
'telomere length || id:ieu−b−4879' on 'IDP_dMRI_TBSS_L2_Superior_fronto−occipital_fascic
